# Prolonged Prophylactic Antibiotics Based on Preoperative Bile Culture Reduce Surgical Site Infections After Pancreaticoduodenectomy Following Preoperative Biliary Drainage: A Propensity‐Matched Analysis

**DOI:** 10.1002/ags3.70076

**Published:** 2025-08-14

**Authors:** Kyohei Matsumoto, Atsushi Shimizu, Yuji Kitahata, Akihiro Takeuchi, Hideki Motobayashi, Masatoshi Sato, Tomohiro Yoshimura, Shinya Hayami, Atsushi Miyamoto, Manabu Kawai

**Affiliations:** ^1^ Second Department of Surgery, School of Medicine Wakayama Medical University Wakayama Japan

**Keywords:** pancreaticoduodenectomy, postoperative complication, preoperative biliary drainage, prophylactic antibiotic, surgical site infections

## Abstract

**Objective:**

The optimum duration of prophylactic antibiotics after pancreaticoduodenectomy following preoperative biliary drainage to prevent surgical site infections remains controversial. We evaluate whether a prolonged course of prophylactic antibiotics reduces surgical site infection after pancreaticoduodenectomy following biliary drainage more than that within the standard duration.

**Methods:**

We enrolled 352 consecutive patients from one hospital who underwent pancreaticoduodenectomy following biliary drainage between 2010 and 2023. The patients were prospectively assigned to two groups according to prophylactic antibiotic duration. In the standard duration group (2010–2013; 112 patients), the duration was within 24 h postoperatively. In the prolonged duration group (2014–2023; 240 patients), it was 3 days postoperatively. The primary endpoint was the incidence of surgical site infection between these groups. We performed 1:1 propensity score matching to balance baseline characteristics, which yielded 77 patients per group.

**Results:**

There was significantly less surgical site infection in the longer duration group (13%) than in the standard duration group (29%) (*p* = 0.0010). After matching, the prolonged duration group maintained significantly lower rates of all surgical site infection (32% vs. 14%, *p* = 0.0126), organ/space surgical site infection (27% vs. 13%, *p* = 0.0433), incisional surgical site infection (18% vs. 3%, *p* = 0.0026), superficial incisional surgical site infection (13% vs. 3%, *p* = 0.0314). In the multivariate analysis, independent risk factors for surgical site infection after pancreaticoduodenectomy following biliary drainage were elevated drain fluid amylase on postoperative day 1 (*p* < 0.0001) and 1‐day prophylactic antibiotics (*p* = 0.00012).

**Conclusions:**

Prolonged prophylactic antibiotics significantly reduced surgical site infection incidence after pancreaticoduodenectomy in patients undergoing preoperative biliary drainage.

## 1 Introduction

Pancreaticoduodenectomy for pancreatic cancer or periampullary tumors carries a high risk of postoperative complications (40%–60%) and mortality rates of 2%–5% [1, 2, 3]. Surgical site infection (SSI) can be severely problematic after pancreaticoduodenectomy [3, 4], leading to prolonged hospital stays and potentially fatal complications such as intra‐abdominal abscess or sepsis [2]. Guidelines for preventing SSI recommend that prophylactic antibiotics be administered intraoperatively or up to 24 h after surgery, with cephalosporins being the most frequently used agents for gastrointestinal procedures, including pancreaticoduodenectomy [5, 6].

Prophylaxis piperacillin–tazobactam, a broader‐spectrum antibiotic, was shown in several recent studies to effectively reduce SSIs not only in patients undergoing pancreaticoduodenectomy [7, 8], but also in those undergoing preoperative biliary drainage followed by pancreaticoduodenectomy [9, 10]. From the perspective of antimicrobial resistance, minimizing the use of prophylactic antibiotics whenever possible to mitigate the risk of antimicrobial resistance is crucial. However, preoperative biliary drainage in patients undergoing pancreaticoduodenectomy reportedly increases the risk of SSI, highlighting the need for advanced strategies for determining the most appropriate perioperative prophylactic antibiotic treatment [11, 12, 13]. Specifically, the optimal duration of prophylactic antibiotics in patients undergoing preoperative biliary drainage followed by pancreaticoduodenectomy currently lacks evidence. However, in the literature, some studies and one meta‐analysis have reported that prolonged prophylactic antibiotics after pancreaticoduodenectomy in such patients significantly reduced major complications such as deep SSI [14, 15, 16, 17]. Establishing the optimal duration of prophylactic antibiotics to prevent SSI after pancreaticoduodenectomy in high‐risk patients with preoperative biliary drainage is thought to be essential to improve patient outcomes and to optimize healthcare resources.

Since 2014, for patients with preoperative bile drainage, we have adopted a strategy of prolonged prophylactic antibiotics treatment. It is selected based on bile culture and drug susceptibility test using bile collected during preoperative drainage to prevent postoperative SSI. Here, we evaluate whether this treatment strategy for prolonged prophylactic antibiotics reduces the incidence of SSI compared with the conventional course of prophylactic antibiotics (within 24 h after surgery) recommended by guidelines [5, 6].

## 2 Patients and Methods

### 2.1 Patient Characteristics

We reviewed a prospectively managed database of patients who underwent pancreatic head resection for peri‐pancreatic disease at our hospital between January 2010 and December 2023. Among the 827 patients who received pancreaticoduodenectomy, 352 patients with preoperative biliary drainage were included in the analysis. To assess the value of prolonged prophylactic antibiotics, we dichotomized the patients according to the period when they were treated. Between 2010 and 2013, 112 patients received prophylactic antibiotics only within 24 h after surgery (the standard duration group). Then, between 2014 and 2023, 240 patients prospectively received prophylactic antibiotics for 3 days postoperatively to evaluate whether the incidence of SSIs could be reduced by prolonged prophylactic antibiotics (the prolonged duration group).

**FIGURE 1:**
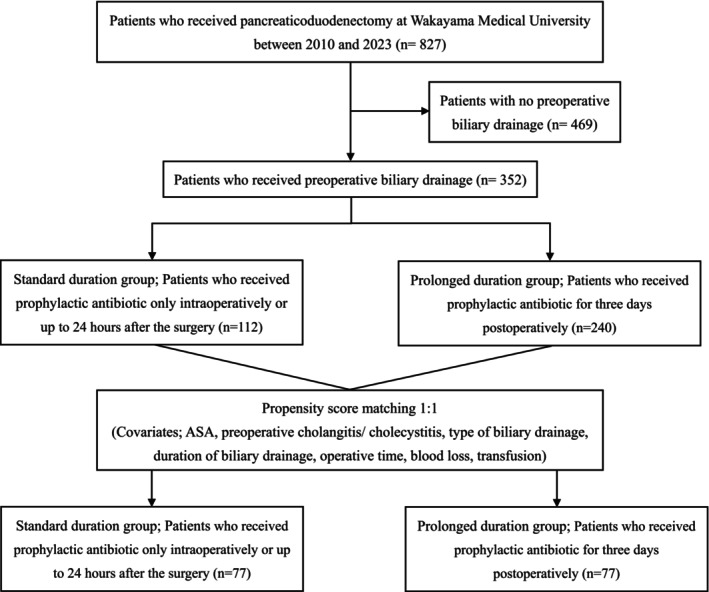
Study flow diagram showing patient selection and grouping.

Propensity score matching (1:1) was performed to balance baseline characteristics between the groups, resulting in 76 patients in each group (Figure 1). This retrospective study was approved by the Wakayama Medical University Hospital Institutional Review Board (Registration No. 4345), and it was registered with the UMIN Clinical Trials Registry (Registration No. UMIN000057002). The protocol for this research project conforms to the provisions of the Declaration of Helsinki. This requirement for individual informed consent was waived for this retrospective study, following the “opt‐out” principle. The patients were allowed to “opt out” of the database if they wished.

### 2.2 Preoperative Management

The methods for preoperative biliary drainage have changed over this study period by improving biliary endoscopic technique or instrument as follows: percutaneous transhepatic biliary drainage, endoscopic nasobiliary drainage, and biliary stent. In the prolonged duration group (the latter group), the use of biliary stent was chosen more frequently due to improvements in biliary endoscopic technique or instrument. The method of preoperative biliary drainage was selected according to preferences by endoscopists and surgeons.

Regarding preoperative treatment for pancreatic cancer, our institution started neoadjuvant therapy for potentially resectable pancreatic cancer or borderline resectable pancreatic cancer with radiologic portal vein/superior mesenteric vein involvement or artery involvement in 2013. However, neoadjuvant therapy regimes have changed over the course of our study period as follows: (i) radiation with concurrent S‐1 (oral 5‐fluoroulracil prodrug tegafur, with oteracil and gimeracil) as chemoradiotherapy, (ii) gemcitabine plus S‐1 therapy, (iii) modified FOLFIRINOX (without bolus 5‐FU and LV, also decreased dose of irinotecan), or (iv) nab‐paclitaxel plus gemcitabine therapy.

### 2.3 Surgical Procedure

During the period of this study, just two pancreatic surgeons with more than 20 years of experience performed pancreaticoduodenectomy until June 2011; after that, an advanced skill specialist certified by the Japanese Society of Hepato–Biliary–Pancreatic Surgery also performed the operation as the surgeon or teaching assistant for all operations. All patients underwent pylorus‐resecting pancreaticoduodenectomy via the antecolic route, with varying extents of lymph node dissection [18]. Reconstruction was performed using the Child method, wherein the elevated jejunal limb was brought through the transverse mesocolon in the retrocolic route. Pancreatojejunostomy was performed using end‐to‐side anastomosis with a single layer of interrupted absorbable stitches. Internal pancreatic duct stents were generally inserted using the duct‐to‐mucosa method to prevent pancreatic fistulas. In seromuscular parenchymal anastomosis, suture of pancreatic parenchyma and jejunal seromuscular layer is performed by the modified Blumgart mattress method. The end‐to‐side hepaticojejunostomy was created 15 cm distal to pancreatojejunostomy on the same limb. No stent was placed at the anastomosis site of the hepaticojejunostomy. Gastrojejunostomy was performed through the antecolic route, and reconstruction was completed. Before closing the abdomen, the abdominal cavity was washed with a total of 5000 mL of warm saline solution. Finally, a single closed‐suction drain was routinely placed around the pancreatojejunostomy site. The nasogastric tube was removed immediately after intratracheal extubation in the operating room [19].

### 2.4 Perioperative Antibiotic and Perioperative Management

Preoperative antibiotic therapy was performed for preoperative biliary drainage or for the treatment of cholangitis. In the case of preoperative biliary drainage for patients with jaundice needing pancreaticoduodenectomy, we intravenously administered 2 g of cefazolin before biliary drainage. As for intraoperative prophylactic antibiotics, either cefazolin (1 g) or flomoxef (1 g) was administered, with the full dose completed within 30 min before the skin incision. Additional doses were repeated every 3 h during pancreaticoduodenectomy. Until 2014, prophylactic antibiotic was administered only intraoperatively or up to 24 h after the surgery as a standardized regimen in accordance with the World Health Organization guidelines [5], and this group is referred to as “standard duration group.” Beginning in 2014, prolonged prophylactic antibiotic therapy for three postoperative days was adopted. The types of postoperative prophylactic antibiotics were selected based on an antibiotic sensitive to the isolated bacteria by bile cultures obtained during preoperative biliary drainage procedures; this group is referred to as “prolonged duration group.” In cases where no specific bacteria were identified from bile cultures, the type of postoperative prophylactic antibiotics in the prolonged duration group was selected according to the surgeon's preference, based on the intraoperative findings and the patient's clinical and laboratory status.

The drain was removed on postoperative day 4 if the drainage fluid was clear, and in the absence of pancreatic fistula and bacterial contamination. Amylase levels in the serum and the drainage fluid were routinely measured on postoperative days 1, 3, and 4 [20]. Proton pump inhibitors were administered intravenously for 3 days after surgery and then orally thereafter. Octreotide prophylaxis was not routinely given.

### 2.5 Study Endpoints

The primary endpoint of this study was the incidence of SSI, including superficial incisional SSI, deep incisional SSI, and organ/space SSI. SSIs were defined as infections developing within 30 days after surgery. The definition and classification of SSI in this study are based on the established criteria [21]. Secondary endpoints included postoperative complications specific to pancreaticoduodenectomy, such as pancreatic fistulas [22], delayed gastric emptying [23], and post‐pancreatectomy hemorrhage [24] according to the criteria of the International Study Group on Pancreatic Surgery. Other postoperative complications were redefined based on the Clavien classification of more than grade II [25]. Severe complications were defined as Clavien grade III or higher. Mortality was defined as death within 90 days of surgery.

### 2.6 Clinical Data

All data were extracted from electronic medical records. Clinical data were collected prospectively for all patients and included patient demographics, pathological examination, perioperative clinical information, and complications. When the gastroenterologist or surgeon diagnosed hepatic dysfunction for tumor‐causing obstructive jaundice in patients for whom pancreaticoduodenectomy was planned, the patients underwent preoperative biliary drainage by percutaneous transhepatic biliary drainage, endoscopic nasobiliary drainage, or biliary stents. For preoperative bile cultures, each patient's bile juice was collected and cultured during preoperative biliary drainage procedures. If biliary drainage had been performed multiple times, we used the most recent bile culture results before surgery.

### 2.7 Statistical Analysis

Data are presented as percentages for categorical variables or as medians with interquartile range (IQR) values for continuous variables. Continuous variables were analyzed using the Mann–Whitney *U* test, nominal variables were analyzed using Fisher's exact test or the Chi‐square test, depending on the sample size and expected frequencies, and ordinal variables were analyzed using the Chi‐square test. Propensity score matching (PSM) was performed using a logistic regression model to adjust for baseline differences between the two groups, with a caliper width of 0.2. A 1:1 nearest‐neighbor matching without replacement was applied. Covariates included ASA‐PS, presence of preoperative cholangitis or cholecystitis, type of preoperative biliary drainage (internal or external), duration of preoperative biliary drainage, operative time, blood loss, and presence of transfusion. Before matching, a total of 352 patients were included in the analysis. After matching, 76 patients were selected in each group. PSM was selected instead of inverse probability of treatment weighting (IPTW) for statistical analysis in this study by the following reasons: (1) The validity of the propensity score model was further supported by the receiver operating characteristic (ROC) curve (Figure S1) derived from the multivariable logistic regression model used to generate the propensity scores, which demonstrated good discriminatory ability (AUC = 0.82). (2) PSM can reduce the problem of extreme weights by excluding patients with very high or low propensity scores during the matching process. In contrast, IPTW can be highly sensitive to individuals with propensity scores near 0 or 1, leading to unstable estimates and increased variance due to large weights. Figure S2 showed there was a limited overlap in the distributions of propensity scores between the two groups. The majority of patients in the prolonged duration group exhibited high propensity scores (> 0.8), whereas those in the standard duration group were predominantly located in the lower range, resulting in minimal overlap. Applying IPTW in this context would necessitate disproportionately large weights for patients in the standard group with high propensity scores, thereby increasing the risk of unstable estimates and inflated variance. IPTW creates a synthetic population by reweighting, which may not reflect the characteristics of any real‐world patient group. (3) Figure S3 showed PSM effectively restricted the analysis to the region of common support and improved covariate balance between the groups. PSM allows for comparisons between actual patients with similar characteristics, leading to a more clinically interpretable and realistic comparison group. Taken together, we believe that PSM was the most appropriate and methodologically sound approach for our dataset.

Univariate analyses were performed prior to multivariable analyses, and all variables were categorized into nominal variables for these analyses. Clinically relevant factors identified in univariate analyses were included in a multivariable logistic regression model to adjust for potential confounding variables and determine independent associations with SSI. A two‐sided *p*‐value of < 0.05 was considered statistically significant, and all analyses were performed using JMP Statistical Discovery software (version 17.0; SAS Institute, Cary, NC, USA).

## 3 Results

### 3.1 Preoperative Patient Characteristics, Operative Variables, and Postoperative Complications, Before and After Propensity Score Matching

Table 1 summarizes the results of the total cohort and the matched cohort after PSM. In the total cohort, comparison with the standard duration group (*n* = 112) showed that the prolonged duration group (*n* = 240) had a significantly higher proportion of patients with ASA‐PS ≥ 3 (*p* < 0.0001), preoperative cholangitis or cholecystitis (*p* = 0.0018), internal biliary drainage (*p* < 0.0001), and a longer duration of preoperative biliary drainage (*p* < 0.0001). The use of biliary stent has significantly increased in the prolonged duration group compared to the standard duration group (the standard duration group 62.5% vs. the prolonged duration group 92.9%, *p* < 0.0001) (Table S1). Only when the biliary stent failed for biliary drainage, PTBD, or ENBD was performed. No EUS‐guided hepaticogastrostomy was used as preoperative biliary drainage in this study. However, the difference in preoperative biliary drainage methods did not technically affect the incidence of SSI in this study (Table S1).

**TABLE 1:** Preoperative patient characteristics, operative variables, and postoperative complications, before and after propensity score matching.

	Total cohort			Matched cohort		
	Standard duration group (*n* = 112)	Prolonged duration group (*n* = 240)	*p*	Standard duration group (*n* = 76)	Prolonged duration group (*n* = 76)	*p*
Patient characteristics						
Age, median (IQR)	71 (63–76)	71 (65–76)	0.9991	71 (62–76)	71 (66–77)	0.4319
Gender (male), *N* (%)	68 (61)	151 (63)	0.7240	44 (58)	42 (55)	0.7434
BMI, median (IQR)	21.9 (20.1–23.9)	22.1 (20.0–24.4)	0.6199	22.3 (20.2–25.2)	22.8 (20.9–25.2)	0.0999
Smoking (yes), *N* (%)	48 (43)	125 (52)	0.1108	32 (42)	35 (46)	0.6241
Alcohol use history (yes), *N* (%)	45 (40)	94 (39)	0.9069	30 (39)	28 (37)	0.7384
Steroid use (yes), *N* (%)	0 (0)	6 (3)	0.1825	0 (0)	2 (3)	0.1546
Diabetes mellitus (yes), *N* (%)	28 (25)	57 (24)	0.7908	20 (26)	16 (21)	0.4454
ASA‐PS (1/2/3), *N* (%)	10 (9)/87 (78)/15 (13)	3 (1)/223 (92)/14 (6)	< 0.0001*	4 (5)/64 (84)/8 (11)	3 (4)/64 (83)/10 (13)	0.8299
Diagnosis (pancreatic cancer), *N* (%)	53 (47)	116 (48)	0.9090	36 (47)	33 (43)	0.6250
NAT (Yes), *N* (%)	10 (9)	46 (19)	0.0144*	9 (12)	8 (11)	0.7969
Preoperative cholangitis/cholecystitis (yes), *N* (%)	22 (20)	88 (37)	0.0013*	18 (24)	17 (22)	0.8472
Type of initial biliary drainage (internal), *N* (%)	70 (63)	223 (93)	< 0.0001*	59 (78)	59 (78)	1.0000
Duration of preoperative biliary drainage, median (IQR)	36 (24–55)	46.5 (32–86)	< 0.0001*	40.5 (24–62)	41 (27.3–66.8)	0.5666
Laboratory data						
HbA1c, median (IQR)	5.6 (5.1–6.5)	5.8 (5.4–6.3)	0.1829	5.7 (5.1–6.4)	5.7 (5.4–6.2)	0.5712
mGPS (0/1/2), *N* (%)	53 (47)/47 (42)/12 (11)	123 (51)/77 (32)/40 (17)	0.1235	39 (51)/31 (41)/6 (8)	40 (53)/20 (26)/16 (21)	0.0313*
PNI, median (IQR)	44.5 (40–52)	42.7 (39.1–47.8)	0.4755	45.3 (40.6–49.1)	43.4 (38.0–48.7)	0.1565
Operative variables						
Operation time, median (IQR)	429 (365–479)	374.5 (327.3–432.8)	0.0004*	418 (345–462)	394.5 (351–454)	0.5383
Blood loss, median (IQR)	450 (282–892.3)	220.5 (116.3–723.5)	< 0.0001*	378 (240–596)	288 (155–709)	0.2412
Transfusion (yes), *N* (%)	21 (19)	11 (5)	< 0.0001*	9 (12)	8 (11)	0.7969
Portal vein resection (yes), *N* (%)	33 (29)	52 (22)	0.1410	19 (25)	16 (21)	0.5633
Main pancreatic duct, median (IQR), *N* (%)	3 (2–4)	3 (2–4)	0.2700	3 (2–4)	3 (2–4)	0.4587
Pancreatic texture (soft), *N* (%)	46 (41)	121 (50)	0.1097	34 (45)	41 (54)	0.2561
Drain fluid AMY level in POD1 (IU/L), median (IQR)	721.5 (134.3–2460.5)	1080.5 (154–3460.3)	0.2596	621 (107–4199)	1210 (92–5097)	0.6570
Post operative complications						
All SSI, *N* (%)	32 (29)	32 (13)	0.0010*	22 (29)	11 (14)	0.0305*
Organ/space SSI, *N* (%)	27 (24)	26 (11)	0.0021*	19 (25)	9 (12)	0.0364*
Incisional SSI, *N* (%)	16 (14)	8 (3)	0.0004*	11 (14)	3 (4)	0.0248*
Superficial incisional SSI, *N* (%)	12 (11)	8 (3)	0.0111*	7 (9)	3 (4)	0.1906
Deep incisional SSI, *N* (%)	5 (4)	2 (1)	0.0356*	4 (5)	1 (1)	0.1725
Severe complications (CD grade III or more), *N* (%)	28 (25)	25 (10)	0.0007*	18 (24)	12 (16)	0.2214
Pancreatic fistula (Grade B or more), *N* (%)	22 (20)	16 (7)	0.0007*	15 (20)	6 (8)	0.0344*
Intra‐abdominal abscess, *N* (%)	27 (24)	24 (10)	0.0010*	19 (25)	9 (12)	0.0364*
Post‐pancreatectomy hemorrhage, *N* (%)	7 (6)	5 (2)	0.0586	3 (4)	3 (4)	1.0000
Delayed gastric emptying, *N* (%)	11 (10)	14 (6)	0.1860	5 (7)	6 (8)	0.7542
Percutaneous drainage, *N* (%)	25 (22)	22 (9)	0.0012*	16 (21)	10 (13)	0.1962
Reoperation, *N* (%)	3 (3)	3 (1)	0.3874	2 (3)	2 (3)	1.0000
Mortality, *N* (%)	1 (1)	0 (0)	0.3182	1 (1)	0 (0)	0.3157

Abbreviations: ASA‐PS, American Society of Anesthesiology physical status; BMI, body mass index; mGPS, modified Glasgow Prognostic Score; NAT, neo‐adjuvant therapy; PNI, Prognostic Nutritional Index; SSI, surgical site infection.

* Statistically significant.

No other factors were significantly different between the two groups. Regarding operative variables, surgeries in the prolonged duration group were associated with shorter operative times (*p* = 0.0004), reduced blood loss (*p* < 0.0001), and a lower transfusion rate (*p* < 0.0001). The proportion of patients undergoing portal vein resection (*p* = 0.1410), those with differences in main pancreatic duct diameter (*p* = 0.3504), and those with soft pancreatic texture (*p* = 0.1097) were similar between the groups. As for postoperative outcomes, the prolonged duration group exhibited lower rates of all SSI (*p* = 0.0010), organ/space SSI (*p* = 0.0021), incisional SSI (*p* = 0.0004), and superficial incisional SSI (*p* = 0.0111) compared with the standard duration group. Furthermore, the prolonged duration group had significantly lower rates of severe complications (Clavien–Dindo grade III or higher; *p* = 0.0007), intra‐abdominal abscess (*p* = 0.0023), and percutaneous drainage (*p* = 0.0012).

After PSM, baseline differences in patient characteristics and operative variables were adjusted, resulting in no significant differences between the matched groups in terms of preoperative or operative factors. Due to differences in historical background, the number of patients who received neoadjuvant therapy for pancreatic cancer significantly increased in the prolonged duration group compared with in the standard duration group (9% vs. 19%, *p* = 0.0144), however, after propensity score matching, no significant difference between the matched groups in terms of neoadjuvant therapy was observed (*p* = 0.8064). Even after this adjustment, postoperative complications, particularly SSI and other severe outcomes, were independently less frequent in the prolonged duration group. Moreover, it continued to show lower rates of overall SSI (*p* = 0.0126), organ/space SSI (*p* = 0.0430), and severe complications (*p* = 0.0279) after adjusting for baseline characteristics. Additionally, the prolonged duration group maintained significantly lower rates of intra‐abdominal abscess (*p* = 0.0433) and percutaneous drainage (*p* = 0.0322) than the standard duration group.

We also evaluated the efficacy of prolonged prophylactic antibiotics in only patients who underwent internal stent as preoperative biliary drainage to prevent the selection bias of preoperative biliary drainage methods (Table S2). As same as the results of total cohort, the prolonged duration group exhibited significantly lower rates of all SSI (*p* = 0.0073), organ/space SSI (*p* = 0.0038), and severe complications (*p* = 0.0280) compared with the standard duration group even after adjusting for baseline characteristics in patients who underwent internal stent as preoperative biliary drainage (Table S2).

### 3.2 Risk Factors for SSI After Pancreaticoduodenectomy

A total of 352 patients were analyzed to evaluate risk factors for SSIs after pancreaticoduodenectomy. Among them, 64 patients (18%) developed SSIs, while 288 patients (82%) did not (Table 2). Both univariate and multivariate analyses were performed to identify significant associations with the occurrence of SSIs. In the univariate analysis, several factors were significantly associated with the occurrence of SSIs. A diagnosis of pancreatic cancer was less frequent in the SSI group compared with the non‐SSI group (OR: 0.55, 95% CI: 0.31–0.96, *p* = 0.0380). Patients with soft pancreatic texture were more likely to develop SSIs than those with hard pancreatic texture (OR: 2.49, 95% CI: 1.41–4.38, *p* = 0.0014). Similarly, a main pancreatic duct diameter of ≤ 3 mm was significantly associated with SSIs, occurring in 53% of the SSI group compared with 36% in the non‐SSI group (OR: 2.00, 95% CI: 1.16–3.46, *p* = 0.0157). Elevated postoperative day 1 drain fluid amylase levels on > 4000 IU/L were strongly associated with SSIs, occurring in 50% of the SSI group compared with 16% of the non‐SSI group (OR: 5.26, 95% CI: 2.94–9.42, *p* < 0.0001). Furthermore, prophylactic antibiotic for 3 days postoperatively was associated with a significantly lower risk of SSIs compared with prophylactic antibiotics within 24 h after surgery (OR: 0.38, 95% CI: 0.22–0.67, *p* = 0.0010).

**TABLE 2:** Risk factors of SSI after PD.

	SSI (*n* = 64) (18%)	No SSI (*n* = 288) (82%)	Univariate analysis			Multivariate analysis		
			OR	95% CI	*p*	OR	95% CI	*p*
Age (≥ 75), *n* (%)	23 (36)	88 (31)	1.27	0.72–2.25	0.4573			
Gender (male), *n* (%)	43 (67)	176 (61)	1.30	0.73–2.31	0.3953			
BMI (≥ 25), *n* (%)	16 (25)	48 (17)	1.67	0.87–3.18	0.1503			
Smoking (yes), *n* (%)	27 (42)	146 (51)	0.71	0.41–1.23	0.2688			
Alcohol use history (yes), *n* (%)	28 (44)	111 (39)	1.24	0.72–2.15	0.4807			
Steroid use (yes), *n* (%)	1 (2)	5 (2)	0.90	0.10–7.82	1.0000			
Diabetes mellitus (yes), *n* (%)	11 (17)	74 (26)	0.60	0.30–1.21	0.1959			
ASA‐PS (≥ 3), *n* (%)	6 (9)	23 (8)	1.19	0.46–3.06	0.8011			
Diagnosis (pancreatic cancer), *n* (%)	23 (36)	146 (51)	0.55	0.31–0.96	0.0380*	1.84	0.70–4.84	0.20415
Preoperative cholangitis/cholecystitis (yes), *n* (%)	19 (30)	91 (32)	0.91	0.51–1.65	0.8816			
Type of initial biliary drainage (internal), *n* (%)	53 (83)	240 (83)	0.96	0.47–1.98	1.0000			
Duration of preoperative biliary drainage (≥ 45 days), *n* (%)	30 (47)	143 (50)	0.89	0.52–1.54	0.7824			
Laboratory data								
HbA1c (≥ 6.5%), *n* (%)	11 (17)	69 (24)	0.66	0.33–1.33	0.3221			
Albumin (≥ 3.5 g/dL), *n* (%)	26 (41)	133 (46)	0.80	0.46–1.38	0.4880			
mGPS (≥ 2), *n* (%)	8 (13)	44 (15)	0.79	0.35–1.78	0.6982			
Operation time (≥ 399 min: average), *n* (%)	32 (50)	132 (46)	1.18	0.69–2.03	0.5811			
Blood loss (≥ 444 mL: average), *n* (%)	25 (39)	90 (31)	1.41	0.81–2.47	0.2407			
Transfusion (yes), *n* (%)	8 (13)	24 (8)	1.57	0.67–3.68	0.3345			
Pancreatic texture (soft), *n* (%)	42 (66)	125 (43)	2.49	1.41–4.38	0.0014*	2.51	0.93–6.81	0.06433
Main pancreatic duct ≤ 3 mm, *n* (%)	34 (53)	104 (36)	2.00	1.16–3.46	0.0157*	0.96	0.49–1.86	0.90030
Drain fluid AMY level in POD1 > 4000 IU/L, *n* (%)	32 (50)	46 (16)	5.26	2.94–9.42	< 0.0001*	5.23	2.59–10.53	< 0.0001*
Prophylactic antibiotic for 3 days, *n* (%)	32 (50)	208 (72)	0.38	0.22–0.67	0.0010*	0.30	0.16–0.56	0.00012*

* Statistically significant.

In the multivariate analysis, two factors emerged as independent risk factors for SSIs. Elevated drain fluid amylase levels on postoperative day 1 > 4000 IU/L remained a significant predictor of SSIs (OR: 5.23, 95% CI: 2.59–10.53, *p* < 0.0001). Additionally, patients treated with prophylactic antibiotics for 3 days postoperatively were independently associated with a significantly lower risk of SSIs (OR: 0.30, 95% CI: 0.16–0.56, *p* = 0.00012).

### 3.3 Risk Factors for SSI After Pancreaticoduodenectomy in Patients With Preoperative Cholangitis/Cholecystitis

We analyzed risk factors for SSI in patients with preoperative cholangitis or cholecystitis. In this cohort, 19 patients (17%) developed SSI, while 91 patients (83%) did not (Table 3). Univariate analysis identified several factors significantly associated with SSI risk. These included a high BMI (*p* = 0.0255), diagnosis other than pancreatic cancer (*p* = 0.0104), prolonged operation time (*p* = 0.0458), soft pancreatic texture (*p* = 0.0098), a thin main pancreatic duct diameter (*p* = 0.0017), and high drain fluid amylase levels on postoperative day 1 (*p* = 0.0002). Additionally, the use of prophylactic antibiotics for 3 days was associated with a reduced risk of SSI (*p* = 0.0226).

**TABLE 3:** Risk factors for SSI after PD in patients with preoperative cholangitis/cholecystitis.

	SSI (*n* = 19) (17%)	No SSI (*n* = 91) (83%)	Univariate analysis			Multivariate analysis		
			OR	95% CI	*p*	OR	95% CI	*p*
Age (≥ 75), *n* (%)	6 (32)	25 (27)	1.28	0.42–3.56	0.7174			
Gender (male), *n* (%)	17 (89)	64 (70)	3.59	0.77–16.6	0.1494			
BMI (≥ 25), *n* (%)	8 (42)	15 (16)	3.68	1.27–10.70	0.0255*	2.40	0.63–9.14	0.20554
Smoking (yes), *n* (%)	13 (68)	53 (58)	1.55	0.45–4.45	0.4526			
Alcohol use history (yes), *n* (%)	13 (68)	43 (47)	2.42	0.85–6.92	0.1302			
Steroid use (yes), *n* (%)	0 (0)	1 (1)	0	N/A	1.0000			
Diabetes mellitus (yes), *n* (%)	4 (21)	21 (23)	0.89	0.27–2.97	1.0000			
ASA‐PS (≥ 3), *n* (%)	3 (16)	8 (9)	1.95	0.47–8.13	0.3992			
Diagnosis (pancreatic cancer), *n* (%)	3 (16)	44 (48)	0.20	0.05–0.73	0.0104*	1.72	0.07–42.01	0.73740
Type of initial biliary drainage (internal), *n* (%)	16 (84)	81 (89)	0.66	0.16–2.66	0.6948			
Duration of preoperative biliary drainage (≥ 45 days), *n* (%)	10 (53)	56 (62)	0.69	0.26–1.88	0.6075			
Laboratory data								
HbA1c (≥ 6.5%), *n* (%)	3 (16)	22 (24)	0.59	0.16–2.21	0.5554			
Albumin (≥ 3.5 g/dL), *n* (%)	9 (47)	45 (49)	0.92	0.34–2.48	1.0000			
mGPS (≥ 2), *n* (%)	3 (16)	17 (19)	0.82	0.21–3.12	1.0000			
Operation time (≥ 399 min: average), *n* (%)	13 (68)	40 (44)	2.76	0.96–7.91	0.0763			
Blood loss (≥ 444 mL: average), *n* (%)	9 (47)	21 (23)	3.00	1.08–8.35	0.0458*	2.89	0.80–10.47	0.10644
Transfusion (yes), *n* (%)	4 (21)	6 (7)	3.78	0.95–15.00	0.0683			
Portal vein resection (yes), *n* (%)	2 (11)	25 (27)	0.31	0.07–1.44	0.1500			
Pancreatic texture (soft), *n* (%)	16 (84)	46 (51)	5.22	1.42–19.14	0.0098*	2.28	0.10–53.32	0.60380
Main pancreatic duct ≤ 3 mm, *n* (%)	4 (21)	57 (63)	6.29	1.93–20.50	0.0017*	2.73	0.60–12.52	0.18543
Drain fluid AMY level in POD1 > 4000 IU/L, *n* (%)	12 (63)	17 (19)	7.46	2.56–21.77	0.0002*	4.70	1.22–18.14	0.01980*
Prophylactic antibiotic for 3 days, *n* (%)	11 (58)	77 (85)	0.25	0.09–0.73	0.0226*	0.39	0.11–1.39	0.14959

* Statistically significant.

Multivariate analysis was performed to adjust for potential confounding factors. High drain fluid amylase levels on postoperative day 1 remained an independent risk factor for SSI (OR: 4.70, 95% CI: 1.22–18.14, *p* = 0.0198). Other factors (the use of prophylactic antibiotics for 3 days, pancreatic texture, and main pancreatic duct diameter) were not independently associated with SSI after adjustment.

### 3.4 Risk Factors for Pancreatic Fistula After Pancreaticoduodenectomy

Both univariate and multivariate logistic regression analyses were performed to evaluate the risk factors for postoperative pancreatic fistula (POPF) (Table S3). In the multivariate analysis, two factors were identified as independent risk factors for POPF as follows: BMI ≥ 25 (OR: 2.81, 95% CI: 1.12–7.87, *p* = 0.0313) and drain amylase level on POD1 > 4000 IU/L (OR: 11.28, 95% CI: 4.30–29.61, *p* < 0.0001). Moreover, prophylactic antibiotic administration for 3 days was significantly associated with a reduced risk of POPF (OR: 0.16, 95% CI: 0.07–0.37, *p* = 0.0001). Similar to the results of risk factors in surgical site infection (SSI) in Table 2 and Table S3 showed prolonged prophylactic antibiotics might also reduce POPF in patients with pancreaticoduodenectomy following preoperative biliary drainage. In the present study, POPF and organ/space SSI were counted separately based on their respective international definitions [21, 22]. However, nearly all POPF grade B or POPF grade C cases were associated with organ/space SSI with positive bacterial cultures from the drain or intra‐abdominal fluid by definitional overlap. As a result, parts of cases with POPF fulfilled the definition of organ/space SSI and were double‐counted as POPF and organ/space SSI.

### 3.5 Microbiological Outcomes

Table 4 showed that the distribution of microorganisms differed significantly between the standard and prolonged antibiotic groups. Polymicrobial infections were more common in the prolonged group (76.5% vs. 52.4%, *p* = 0.0001). In addition, *Enterobacter* spp. was detected in 26.3% of the prolonged group compared to 15.5% in the standard group (*p* = 0.0393), *Escherichia coli* in 16.3% versus 4.8% (*p* = 0.0077), *Klebsiella* spp. in 32.6% versus 15.5% (*p* = 0.0029), *Streptococcus* spp. in 23.9% versus 3.6% (*p* = 0.0001), and *Candida* spp. in 28.5% versus 7.1% (*p* = 0.0001). Table 5 shows the antibiotic susceptibility profiles of organisms isolated from intraoperative bile cultures. *Enterobacter* spp. had significantly lower susceptibility to ampicillin in the prolonged group (6% [2/35]) compared to the standard group (35% [6/17], *p* = 0.0087). For *Enterococcus* spp., susceptibility to meropenem was 12% (6/51) in the standard group and 0% (0/148) in the prolonged group (*p* = 0.0009), while vancomycin susceptibility was 94% versus 99% (*p* = 0.0478). *Escherichia coli* showed higher levofloxacin susceptibility in the prolonged group (83% vs. 0%, *p* = 0.0024), and *Staphylococcus* or *Streptococcus* spp. also had higher levofloxacin susceptibility (77% vs. 44%, *p* = 0.0497). Regarding cefotaxime and cefepime, *Enterococcus* spp. showed low susceptibility in both groups (CTX: 12%–14%, CFPM: 12%–14%), and *Staphylococcus* or *Streptococcus* spp. showed susceptibility to CTX of 22%–53% and to CFPM of 56%–84%.

**TABLE 4:** Distribution of microorganisms isolated from intraoperative bile cultures.

Organism	Standard duration group	Prolonged duration group	*p*
	*n* = 84	*n* = 221	
Single bacterial strain	30 (35.7%)	35 (15.8%)	0.0002
Multiple bacterial strains	44 (52.4%)	169 (76.5%)	0.0001
Gram‐negative bacilli, *n* (%)			
*Acinetobacter baumannii*	1 (1.2%)	3 (1.4%)	
*Aeromonas caviae*	1 (1.2%)	5 (2.3%)	
*Aeromonas hydrophila*	0 (0%)	5 (2.3%)	
*Enterobacter* spp.	13 (15.5%)	59 (26.3%)	0.0393
*Enterobacter aerogenes*	3 (3.6%)	17 (7.7%)	
*Enterobacter cloacae*	10 (11.9%)	42 (19.0%)	
*Escherichia coli*	4 (4.8%)	36 (16.3%)	0.0077
*Klebsiella* spp.	13 (15.5%)	72 (32.6%)	0.0029
*Klebsiella oxytoca*	5 (6.0%)	28 (12.7%)	
*Klebsiella pneumoniae*	8 (9.5%)	37 (16.7%)	
*Klebsiella variicola*	0 (0%)	7 (3.2%)	
*Pseudomonas aeruginosa*	8 (9.5%)	7 (3.2%)	0.0218
*Citrobacter freundii*	11 (13.1%)	21 (9.5%)	0.3603
*Morganella morganii*	1 (1.2%)	4 (1.8%)	0.7035
*Serratia marescens*	2 (2.4%)	6 (2.7%)	0.8705
Gram‐positive cocci, *n* (%)			
*Enterococcus* spp.	52 (62%)	150 (67.9%)	0.3248
*Enterococcus avium*	2 (2.4%)	2 (0.9%)	
*Enterococcus casseliflavus*	3 (3.6%)	13 (5.9%)	
*Enterococcus faecalis*	25 (29.8%)	77 (34.8%)	
*Enterococcus faecium*	19 (22.6%)	48 (21.7%)	
*Enterococcus gallinarum*	2 (2.4%)	3 (1.4%)	
*Enterococcus raffinosus*	1 (1.2%)	7 (3.2%)	
*Staphylococcus* spp.	6 (7.2%)	21 (9.5%)	0.5170
*Staphylococcus aureus*	2 (2.4%)	13 (5.9%)	
*Staphylococcus capitis*	1 (1.2%)	0 (0%)	
*Staphylococcus epidermidis*	3 (3.6%)	8 (3.6%)	
*Streptococcus* spp.	3 (3.6%)	53 (23.9%)	0.0001
*Streptococcus anginosus*	1 (1.2%)	39 (17.6%)	
*Streptococcus oralis*	1 (1.2%)	2 (0.9%)	
*Streptococcus parasanguinis*	0 (0%)	6 (2.7%)	
*Streptococcus sanguis*	1 (1.2%)	6 (2.7%)	
*Candida* spp., *n* (%)	6 (7.1%)	63 (28.5%)	0.0001
*Candida albicans*	6 (7.1%)	36 (16.3%)	
*Candida grabrata*	0 (0%)	27 (12.2%)	
Other	0 (0%)	51 (23.1%)	0.0001

**TABLE 5:** Association between intraoperative bile culture result and antibiotic susceptibility.

	ABPC	PIPC/TAZ	CTX	CFPM	MEPM	VCM	LVFX
*Citrobacter* spp. (*p* value) Standard duration group (*n* = 11) Prolonged duration group (*n* = 23)	0.6379 1 (9) 5 (22)	1.0000 11 (100) 22 (96)	0.2999 8 (73) 21 (91)	— 11 (100) 23 (100)	— 11 (100) 23 (100)	— 0 (0) 0 (0)	1.0000 11 (100) 22 (96)
*Enterobacter* spp. (*p*‐value) Standard duration group (*n* = 14) Prolonged duration group (*n* = 61)	0.0422 3 (21) 2 (3)	1.0000 14 (100) 57 (93)	1.0000 11 (79) 47 (77)	0.5864 14 (100) 55 (90)	— 14 (100) 61 (100)	— 0 (0) 0 (0)	0.3106 12 (86) 57 (93)
*Enterococcus* spp. (*p‐*value) Standard duration group (*n* = 51) Prolonged duration group (*n* = 148)	0.2866 37 (73) 118 (80)	0.3382 37 (73) 117 (79)	0.6628 6 (12) 21 (14)	0.6628 6 (12) 21 (14)	0.0001 6 (12) 0 (0)	0.0223 48 (94) 147 (99)	0.1609 37 (73) 121 (82)
*Escherichia* spp. (*p*‐value) Standard duration group (*n* = 4) Prolonged duration group (*n* = 36)	0.0054 0 (0) 28 (78)	0.3555 3 (75) 33 (92)	0.4925 3 (75) 31 (86)	0.4925 3 (75) 31 (86)	0.1000 3 (75) 36 (100)	— 0 (0) 0 (0)	0.0023 0 (0) 30 (83)
*Klebsiella* spp. (*p*‐value) Standard duration group (*n* = 14) Prolonged duration group (*n* = 69)	0.5958 0 (0) 7 (10)	1.0000 13 (93) 65 (94)	0.5831 14 (100) 64 (93)	1.0000 14 (100) 66 (96)	— 14 (100) 69 (100)	— 0 (0) 0 (0)	1.0000 14 (100) 65 (94)
*Staphylococcus* or *Streptococcus* spp. (*p*‐value) Standard duration group (*n* = 9) Prolonged duration group (*n* = 83)	0.1246 4 (44) 60 (72)	0.4023 6 (67) 66 (80)	0.1868 6 (67) 70 (84)	0.0570 5 (56) 70 (84)	0.0570 5 (56) 70 (84)	— 9 (100) 83 (100)	0.0486 4 (44) 64 (77)

Abbreviations: ABPC, ampicillin; CFPM, cefepime; CTX, cefotaxime; LVFX, levofloxacin; MEPM, meropenem; PIPC/TAZ, piperacillin/tazobactam; VCM, vancomycin.

## 4 Discussion

Prolonged prophylactic antibiotics for 3 days postoperatively were demonstrated by our results to significantly reduce the incidence of SSI after pancreaticoduodenectomy, including severe complications in patients undergoing preoperative biliary drainage, compared with standard‐duration prophylactic antibiotics.

Two outstanding issues to resolve regarding the administration of prophylactic antibiotics after pancreaticoduodenectomy are the duration and type of prophylactic antibiotics.

The optimal duration of prophylactic antibiotics has been unclear. We hypothesized that prolonged postoperative prophylactic antibiotics for 3 days might reduce SSI after pancreaticoduodenectomy in patients with biliary drainage compared with the standard duration of within 24 h after surgery. Some guidelines for standard prophylactic antibiotic regimens actually recommend against prophylactic antibiotics prolonged beyond 24 h after abdominal surgery [5, 6, 21]. However, in several studies, intraoperative positive bile cultures due to preoperative biliary drainage led to a significant increase in SSI or pancreatic fistula after pancreaticoduodenectomy [26, 27]. This emphasized the need for robust prevention strategies. In our previous study, internal biliary drainage and cholangitis before surgery were shown to be associated with an increase in CD grade ≥ III complications [28]. Elsewhere, retrospective studies have reported that high‐risk groups with preoperative biliary drainage require 3–5 days of prolonged postoperative antibiotic therapy to prevent infectious complications [14, 15, 16, 29]. These findings imply that standard prophylactic antibiotic regimens within 24 h after surgery may be insufficient for pancreaticoduodenectomy cases with preoperative biliary drainage. Conversely, a randomized controlled trial reported that prolonged prophylactic cefozopran for 5 days postoperatively did not reduce the incidence of overall infectious complications compared with 1‐day prophylactic use of cefozopran in patients who undergo pancreaticoduodenectomy following preoperative biliary drainage without preoperative cholangitis [30]. The most appropriate duration of postoperative prophylactic antibiotics treatment after pancreaticoduodenectomy in patients undergoing preoperative biliary drainage still lacks consensus: 3, 5, or 7 days [17].

Regarding types of antimicrobial prophylaxis for patients undergoing pancreaticoduodenectomy, one study reported the utility of using piperacillin–tazobactam instead of cefazolin for intraoperative prophylaxis to reduce SSIs following pancreaticoduodenectomy [8]. However, broad‐spectrum antibiotics like piperacillin–tazobactam might permit the emergence of antibiotic‐resistant bacteria and could result in the associated complexities in infection control after pancreaticoduodenectomy. Prior use of third‐generation cephalosporins is also a known risk factor for extended‐spectrum beta‐lactamase‐producing gram‐negative infections [9]. Our results of intraoperative bile cultures state two important findings. First, a longer duration of preoperative biliary drainage might increase polymicrobial infections and the likelihood of biliary colonization by diverse and potentially resistant microorganisms. Second, a considerable number of antibiotic‐resistant organisms were detected in intraoperative bile cultures. Preoperative biliary drainage might facilitate the development or introduction of resistant bacteria into the biliary tract. Taken together, these findings indicate that preoperative biliary drainage can provide information on the presence and antibiotic sensitivity of bacteria in the bile can help select and early administration of appropriate antibiotics, which is considered to be the main source of infection in pancreaticoduodenectomy [31, 32]. We can select the prophylactic antibiotics based on the results of bile culture and drug sensitivity obtained during preoperative biliary drainage.

Our multivariate analysis identified that drain fluid amylase levels > 4000 IU/L on postoperative day 1 and prolonged prophylactic antibiotics were independent predictive factors for SSI after pancreaticoduodenectomy. However, preoperative cholangitis was not associated with SSI after pancreaticoduodenectomy. Preoperative cholangitis reportedly significantly increased ascitic bacterial contamination, and previous studies have reported the incidence of postoperative infectious complications after pancreaticoduodenectomy [28, 33, 34]. The discrepancy may be due to the change to a prolonged prophylactic antibiotics regimen. In the present study, the frequency of preoperative cholangitis in the prolonged duration group was significantly higher than that in the standard duration group (20% vs. 37%, *p* = 0.013). Due to differences in historical background, the number of patients who received neoadjuvant therapy for pancreatic cancer increased in the prolonged duration group compared with the standard duration group. The period from preoperative biliary drainage to surgery was longer in the prolonged duration group, and the frequency of preoperative cholangitis was significantly higher. However, the prolonged prophylactic antibiotics regimen might have prevented SSI due to preoperative cholangitis, even if preoperative cholangitis frequently occurred in the prolonged duration group.

This study had some limitations regarding historical bias and selection bias, is the absence of standardized criteria for antibiotic selection. First, due to the retrospective nature of the study, there were notable differences in baseline characteristics, such as preoperative and operative factors, between the groups that may have influenced these results. We fully acknowledge the possibility of historical bias due to the long study period and the associated improvements in surgical technique and devices. However, we could not include objective measures of surgical skill or institutional experience in the statistical model. We therefore performed PSM to adjust for these factors and to ensure comparability between the groups, reducing the bias of patient characteristics. Even after this adjustment with PSM, prolonged prophylactic antibiotics resulted in lower rates of all SSI, organ/space SSI, severe complications, intra‐abdominal abscess, and percutaneous drainage. These findings might reinforce the association between prolonged prophylactic antibiotics and reduced postoperative complications, independent of baseline differences. A second limitation of this study included the absence of standardized criteria for antibiotic selection, as the choice of antibiotics was determined by surgeons based on bile culture and sensitivity results. From the result of our previous study [28] that preoperative cholangitis significantly increased the incidence of postoperative infectious complications after pancreaticoduodenectomy, we hypothesized that prolonged prophylactic antibiotics would prevent SSI after pancreaticoduodenectomy. Therefore, prophylactic antibiotics for 3 days postoperatively have been started since 2014 for patients with pancreaticoduodenectomy following preoperative biliary drainage. However, this historical cohort with differing backgrounds might make it challenging to convincingly clarify the efficacy of prolonged prophylactic antibiotics from a methodological standpoint. To enhance generalizability, we suggest that clear guidelines for antibiotic selection are necessary. Therefore, we are now planning the phase III randomized controlled trial to evaluate the efficacy of prolonged prophylactic antibiotics after pancreaticoduodenectomy following preoperative biliary drainage.

In conclusion, prolonged prophylactic antibiotics significantly reduced SSI after pancreaticoduodenectomy in patients undergoing preoperative biliary drainage compared with standard duration of prophylactic antibiotics. Our prolonged antibiotic treatment strategy, which involved selecting antibiotics based on preoperative bile culture and sensitivity and administering them for 3 days postoperatively, was suggested to have played a crucial role in reducing postoperative complications. Large‐scale phase III randomized controlled trials are nonetheless required for confirmation. Ultimately, this may lead to wider establishment of postoperative management in patients undergoing pancreaticoduodenectomy with preoperative biliary drainage.

## Author Contributions

**Kyohei Matsumoto:** conceptualization, methodology, software, data curation, investigation, formal analysis, writing – original draft. **Atsushi Shimizu:** writing – review and editing, data curation, conceptualization. **Yuji Kitahata:** writing – review and editing, data curation, conceptualization. **Akihiro Takeuchi:** writing – review and editing, data curation, conceptualization. **Hideki Motobayashi:** writing – review and editing, data curation, conceptualization. **Masatoshi Sato:** writing – review and editing, data curation, conceptualization. **Tomohiro Yoshimura:** writing – review and editing, data curation, conceptualization. **Shinya Hayami:** writing – review and editing, data curation, conceptualization. **Atsushi Miyamoto:** writing – review and editing, data curation, conceptualization. **Manabu Kawai:** conceptualization, methodology, software, data curation, supervision, formal analysis, investigation, writing – original draft, writing – review and editing.

## Ethics Statement

Approval of the research protocol by an Institutional Review Board: This study was approved by the Wakayama Medical University Hospital Institutional Review Board (Registration No. 4345), and it was registered with the UMIN Clinical Trials Registry (Registration No. UMIN000057002).

## Consent

This requirement for individual informed consent was waived for this retrospective study following the “opt‐out” principle. The patients were allowed to “opt out” of the database if they wished.

## Conflicts of Interest

The authors declare no conflicts of interest.

## Supporting information


**Figure S1:** Receiver operating characteristic (ROC) curve for the multivariable logistic regression model predicting prolonged duration group versus standard duration.

**Figure S2:** Violin plot illustrating the distribution of propensity scores in the total cohort.

**Figure S3:** Violin plots illustrating the distribution of propensity scores in the matched and unmatched cohorts.

**Table S1:** Type of preoperative biliary drainage and rate of SSI.

**Table S2:** Preoperative characteristics, operative variables, and post operative complications in patients with internal stent.

**Table S3:** Risk factors of postoperative pancreatic fistula after pancreaticoduodenectomy.

## Data Availability

The datasets used and analyzed during the current study are available from the corresponding author on reasonable request.

## References

[1] J. D. Beane , J. D. Borrebach , A. H. Zureikat , E. M. Kilbane , V. M. Thompson , and H. A. Pitt , “Optimal Pancreatic Surgery: Are We Making Progress in North America?,” Annals of Surgery 274 (2021): e355–e363. 31663969 10.1097/SLA.0000000000003628

[2] F. J. Smits , M. E. Verweij , L. A. Daamen , et al., “Impact of Complications After Pancreatoduodenectomy on Mortality, Organ Failure, Hospital Stay, and Readmission,” Annals of Surgery 275 (2022): e222–e228. 32502075 10.1097/SLA.0000000000003835

[3] A. Pugalenthi , M. Protic , M. Gonen , et al., “Postoperative Complications and Overall Survival After Pancreaticoduodenectomy for Pancreatic Ductal Adenocarcinoma,” Journal of Surgical Oncology 113 (2016): 188–193. 26678349 10.1002/jso.24125 PMC4830358

[4] S. A. M. Karim , K. S. Abdulla , Q. H. Abdulkarim , and F. H. Rahim , “The Outcomes and Complications of Pancreaticoduodenectomy (Whipple Procedure): Cross Sectional Study,” International Journal of Surgery 52 (2018): 383–387. 29438817 10.1016/j.ijsu.2018.01.041

[5] D. J. Leaper and C. E. Edmiston , “World Health Organization: Global Guidelines for the Prevention of Surgical Site Infection,” Journal of Hospital Infection 95 (2017): 135–136. 28139389 10.1016/j.jhin.2016.12.016

[6] D. W. Bratzler , E. P. Dellinger , K. M. Olsen , et al., “Clinical Practice Guidelines for Antimicrobial Prophylaxis in Surgery,” American Journal of Health‐System Pharmacy 70 (2013): 195–283. 23327981 10.2146/ajhp120568

[7] M. De Pastena , S. Paiella , A. M. Azzini , et al., “Antibiotic Prophylaxis With Piperacillinetazobactam Reduces Post‐Operative Infectious Complication After Pancreatic Surgery: An Interventional, Non Randomized Study,” Surgical Infections 22 (2021): 536–542. 33095107 10.1089/sur.2020.260

[8] M. I. D'Angelica , R. J. Ellis , J. B. Liu , et al., “Piperacillin‐Tazobactam Compared With Cefoxitin as Antimicrobial Prophylaxis for Pancreatoduodenectomy: A Randomized Clinical Trial,” JAMA 329 (2023): 1579–1588. 37078771 10.1001/jama.2023.5728 PMC10119777

[9] K. C. Hung , S. J. Chung , A. L. Kwa , W. H. L. Lee , Y. X. Koh , and B. K. P. Goh , “Surgical Prophylaxis in Pancreatoduodenectomy: Is Cephalosporin Still the Drug of Choice in Patients With Biliary Stents In Situ?,” Pancreatology 24 (2024): 960–965. 39068117 10.1016/j.pan.2024.07.004

[10] O. Degrandi , E. Buscail , S. Martellotto , et al., “Perioperative Antibiotherapy Should Replace Prophylactic Antibiotics in Patients Undergoing Pancreaticoduodenectomy Preceded by Preoperative Biliary Drainage,” Journal of Surgical Oncology 120 (2019): 639–645. 31297827 10.1002/jso.25622

[11] Z. V. Fong , M. T. McMillan , G. Marchegiani , et al., “Discordance Between Perioperative Antibiotic Prophylaxis and Wound Infection Cultures in Patients Undergoing Pancreaticoduodenectomy,” JAMA Surgery 151 (2016): 432–439. 26720272 10.1001/jamasurg.2015.4510

[12] L. O. Schoeniger and D. C. Linehan , “Wound Infections After Pancreaticoduodenectomy,” JAMA Surgery 151 (2016): 440. 26720006 10.1001/jamasurg.2015.4659

[13] K. Ohgi , T. Sugiura , Y. Yamamoto , Y. Okamura , T. Ito , and K. Uesaka , “Bacterobilia May Trigger the Development and Severity of Pancreatic Fistula After Pancreatoduodenectomy,” Surgery 160 (2016): 725–730. 27233637 10.1016/j.surg.2016.03.032

[14] M. Steffani , C. Jäger , N. Hüser , et al., “Postoperative Prophylactic Antibiotic Therapy After Pancreaticoduodenectomy in Bile Duct‐Stented Patients Reduces Postoperative Major Complications,” Surgery 176 (2024): 1162–1168. 38769037 10.1016/j.surg.2024.03.025

[15] D. H. M. Droogh , J. L. van Dam , J. V. Groen , et al., “Prolonged Antibiotics After Pancreatoduodenectomy Reduce Abdominal Infections in Patients With Positive Bile Cultures: A Dual‐Center Cohort Study,” HPB: The Official Journal of the International Hepato Pancreato Biliary Association 25 (2023): 1056–1064. 37268503 10.1016/j.hpb.2023.05.008

[16] M. Fromentin , J. Mullaert , B. Gille , et al., “Extended Antibiotic Prophylaxis After Pancreatoduodenectomy Reduces Postoperative Abdominal Infection in High‐Risk Patients: Results From a Retrospective Cohort Study,” Surgery 172 (2022): 205–211. 35140033 10.1016/j.surg.2021.12.028

[17] D. H. M. Droogh , J. V. Groen , M. G. J. de Boer , et al., “Prolonged Antibiotic Prophylaxis After Pancreatoduodenectomy: Systematic Review and Meta‐Analysis,” British Journal of Surgery 110 (2023): 1458–1466. 37440361 10.1093/bjs/znad213 PMC10564402

[18] M. Kawai , M. Tani , S. Hirono , et al., “Pylorus Ring Resection Reduces Delayed Gastric Emptying in Patients Undergoing Pancreatoduodenectomy: A Prospective, Randomized, Controlled Trial of Pylorus‐Resecting Versus Pylorus‐Preserving Pancreatoduodenectomy,” Annals of Surgery 253 (2011): 495–501. 21248633 10.1097/SLA.0b013e31820d98f1

[19] M. Miyazawa , M. Kawai , S. Hirono , et al., “Previous Upper Abdominal Surgery Is a Risk Factor for Nasogastric Tube Reinsertion After Pancreaticoduodenectomy,” Surgery 170 (2021): 1223–1230. 33958204 10.1016/j.surg.2021.03.059

[20] M. Kawai , M. Tani , H. Terasawa , et al., “Early Removal of Prophylactic Drains Reduces the Risk of Intra‐Abdominal Infections in Patients With Pancreatic Head Resection: Prospective Study for 104 Consecutive Patients,” Annals of Surgery 244 (2006): 1–7. 16794381 10.1097/01.sla.0000218077.14035.a6 PMC1570595

[21] Centers for Disease Control and Prevention , “Surgical Site Infection (SSI) Event. CDC Procedure‐Associated Module,” accessed January 25, 2024, https://www.cdc.gov/nhsn/pancreaticoduodenectomyfs/pscmanual/9pscssicurrent.pancreaticoduodenectomyf.

[22] C. Bassi , G. Marchegiani , C. Dervenis , et al., “The 2016 Update of the International Study Group (ISGPS) Definition and Grading of Postoperative Pancreatic Fistula: 11 Years After,” Surgery 161 (2017): 584–591. 28040257 10.1016/j.surg.2016.11.014

[23] M. N. Wente , C. Bassi , C. Dervenis , et al., “Delayed Gastric Emptying After Pancreatic Surgery: A Suggested Definition by the International Study Group of Pancreatic Surgery (ISGPS),” Surgery 142 (2007): 761–768. 17981197 10.1016/j.surg.2007.05.005

[24] M. N. Wente , J. A. Veit , C. Bassi , et al., “Postpancreatectomy Hemorrhage (PPH): An International Study Group of Pancreatic Surgery (ISGPS) Definition,” Surgery 142 (2007): 20–25. 17629996 10.1016/j.surg.2007.02.001

[25] D. Dindo , N. Demartines , and P. A. Clavien , “Classification of Surgical Complications: A New Proposal With Evaluation in a Cohort of 6336 Patients and Results of a Survey,” Annals of Surgery 240 (2004): 205–213. 15273542 10.1097/01.sla.0000133083.54934.ae PMC1360123

[26] K. Nakamura , M. Sho , S. Kinoshita , et al., “New Insight Into the Association Between Bile Infection and Clinically Relevant Pancreatic Fistula in Patients Undergoing Pancreatoduodenectomy,” Journal of Hepato‐Biliary‐Pancreatic Sciences 27 (2020): 992–1001. 32506812 10.1002/jhbp.781

[27] F. Scheufele , L. Aichinger , C. Jäger , et al., “Effect of Preoperative Biliary Drainage on Bacterial Flora in Bile of Patients With Periampullary Cancer,” British Journal of Surgery 104 (2017): e182–e188. 28121036 10.1002/bjs.10450

[28] Y. Kitahata , M. Kawai , M. Tani , et al., “Preoperative Cholangitis During Biliary Drainage Increases the Incidence of Postoperative Severe Complications After Pancreaticoduodenectomy,” American Journal of Surgery 208 (2014): 1–10. 24530042 10.1016/j.amjsurg.2013.10.021

[29] A. Y. Hammad , H. H. Khachfe , S. AlMasri , et al., “Impact of Extended Antibiotic Use After Pancreaticoduodenectomy for Patients With Preoperative Metallic Biliary Stenting Treated With Neoadjuvant Chemotherapy,” Journal of Gastrointestinal Surgery 27 (2023): 716–723. 36650416 10.1007/s11605-023-05581-4 PMC11234506

[30] T. Yamamoto , S. Satoi , T. Fujii , et al., “Dual‐Center Randomized Clinical Trial Exploring the Optimal Duration of Antimicrobial Prophylaxis in Patients Undergoing Pancreaticoduodenectomy Following Biliary Drainage,” Annals of Gastroenterological Surgery 2 (2018): 442–450. 30460348 10.1002/ags3.12209 PMC6236101

[31] R. J. Ellis , B. C. Brajcich , K. A. Bertens , et al., “Association Between Biliary Pathogens, Surgical Site Infection, and Pancreatic Fistula: Results of a Randomized Trial of Perioperative Antibiotic Prophylaxis in Patients Undergoing Pancreatoduodenectomy,” Annals of Surgery 278 (2023): 310–319. 37314221 10.1097/SLA.0000000000005955 PMC10838195

[32] K. Okamura , K. Tanaka , T. Miura , et al., “Randomized Controlled Trial of Perioperative Antimicrobial Therapy Based on the Results of Preoperative Bile Cultures in Patients Undergoing Biliary Reconstruction,” Journal of Hepato‐Biliary‐Pancreatic Sciences 24 (2017): 382–393. 28371248 10.1002/jhbp.453

[33] E. P. Darnell , T. J. Wang , M. A. Lumish , et al., “Preoperative Cholangitis Is an Independent Risk Factor for Mortality in Patients After Pancreatoduodenectomy for Pancreatic Cancer,” American Journal of Surgery 221 (2021): 134–140. 32847686 10.1016/j.amjsurg.2020.07.025

[34] M. Akashi , Y. Nagakawa , Y. Hosokawa , et al., “Preoperative Cholangitis Is Associated With Increased Surgical Site Infection Following Pancreaticoduodenectomy,” Journal of Hepato‐Biliary‐Pancreatic Sciences 27 (2020): 640–647. 32506646 10.1002/jhbp.783

