# Supplementary figures and images for: Prolonged Prophylactic Antibiotics Based on Preoperative Bile Culture Reduce Surgical Site Infections After Pancreaticoduodenectomy Following Preoperative Biliary Drainage: A Propensity‐Matched Analysis
Source: Ann Gastroenterol Surg. 2025 Aug 14;10(1):197–210. doi: 10.1002/ags3.70076 (PMC12757157; doi:10.1002/ags3.70076)

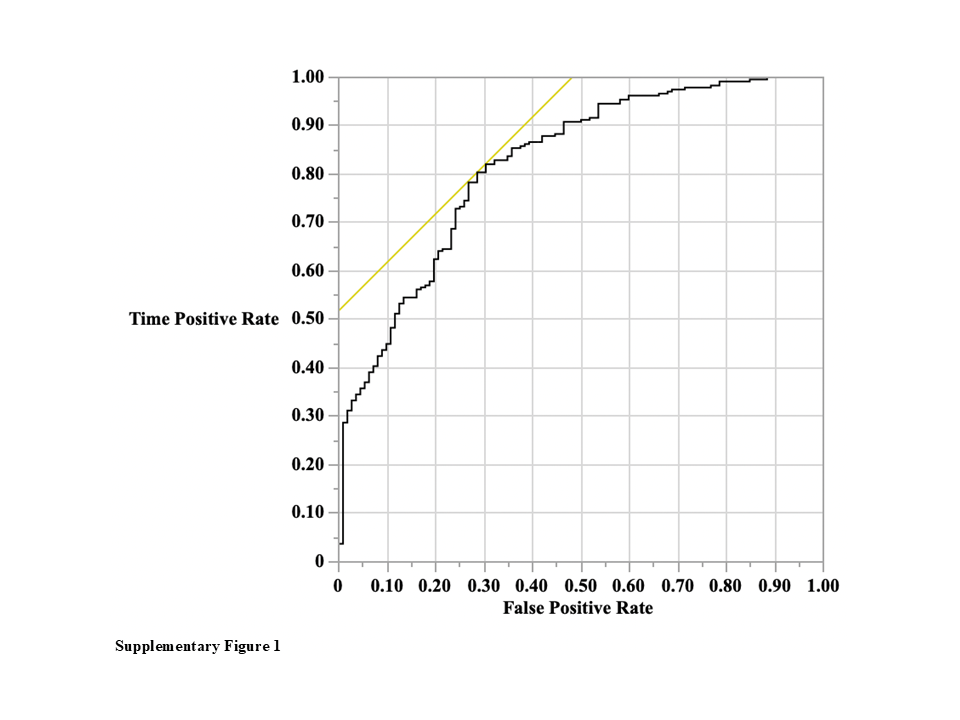

Supplement: Supplementary file 1 — Figure S1: Receiver operating characteristic (ROC) curve for the multivariable logistic regression model predicting prolonged duration group versus standard duration. [file AGS3-10-197-s002.tif]

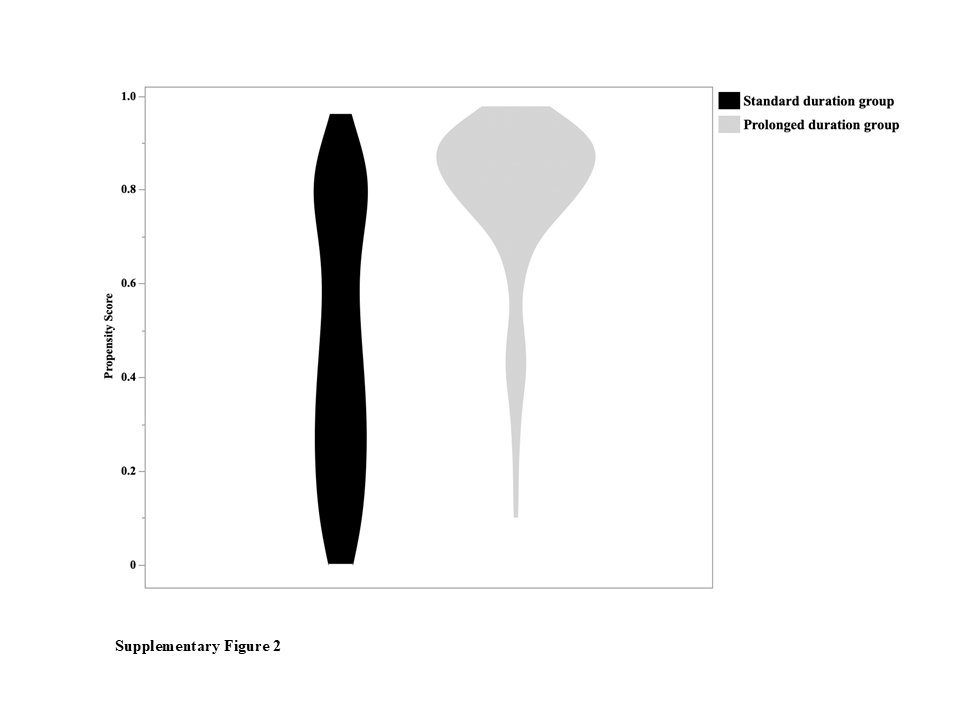

Supplement: Supplementary file 2 — Figure S2: Violin plot illustrating the distribution of propensity scores in the total cohort. [file AGS3-10-197-s003.tif]

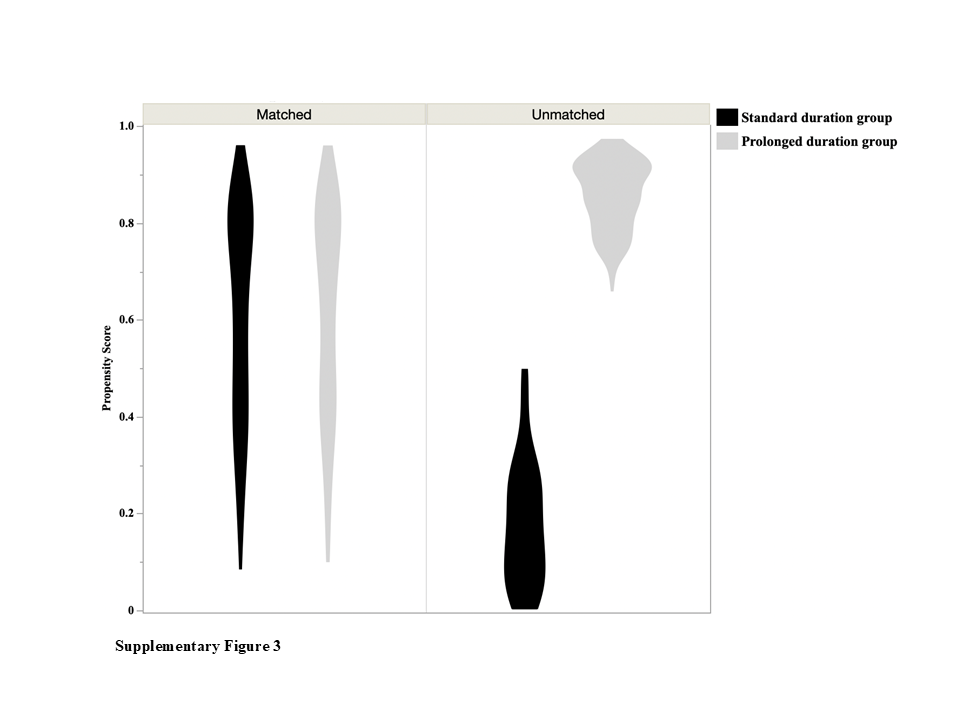

Supplement: Supplementary file 3 — Figure S3: Violin plots illustrating the distribution of propensity scores in the matched and unmatched cohorts. [file AGS3-10-197-s005.tif]
