# Supplementary material for: Prolonged Prophylactic Antibiotics Based on Preoperative Bile Culture Reduce Surgical Site Infections After Pancreaticoduodenectomy Following Preoperative Biliary Drainage: A Propensity‐Matched Analysis
Source: Ann Gastroenterol Surg. 2025 Aug 14;10(1):197–210. doi: 10.1002/ags3.70076 (PMC12757157; doi:10.1002/ags3.70076)
Supplement: Supplementary file 4 — Table S1: Type of preoperative biliary drainage and rate of SSI. [file AGS3-10-197-s004.docx]

**Supplementary Table** **1**

Type of preoperative biliary drainage and rate of SSI

|  | Standard duration group  n=112 | Prolonged duration group  n=240 | *p* value | SSI | p value |
| --- | --- | --- | --- | --- | --- |
| PTBD | 19 (17.0%) | 8 (3.3%) | <0.0001 | 8/27 (29.6%) | 0.1321 |
| ENBD | 23 (20.5%) | 9 (3.8%) |  | 3/32 (9.4%) |  |
| Biliary stent | 70 (62.5%) | 223 (92.9%) |  | 53/293 (18.1%) |  |

PTBD: percutaneous transhepatic biliary drainage; ENBD: endoscopic nasobiliary drainage
