# Supplementary material for: Prolonged Prophylactic Antibiotics Based on Preoperative Bile Culture Reduce Surgical Site Infections After Pancreaticoduodenectomy Following Preoperative Biliary Drainage: A Propensity‐Matched Analysis
Source: Ann Gastroenterol Surg. 2025 Aug 14;10(1):197–210. doi: 10.1002/ags3.70076 (PMC12757157; doi:10.1002/ags3.70076)
Supplement: Supplementary file 5 — Table S2: Preoperative characteristics, operative variables, and post operative complications in patients with internal stent. [file AGS3-10-197-s006.docx]

**Supplementary Table** **2**

Preoperative characteristics, operative variables, and post operative complications in patients with internal stent

|  | Total cohort |  |  | Matched cohort |  |  |
| --- | --- | --- | --- | --- | --- | --- |
|  | standard duration group  (n=70) | prolonged duration group  (n=223) | *p* value | standard duration group  (n=64) | prolonged duration group  (n=64) | *p* value |
| **Patient characteristics** |  |  |  |  |  |  |
| Age, median (IQR) | 70 (63- 77) | 71 (65- 76) | 0.6846 | 70 (63- 76.5) | 71.5 (66.3- 76.8) | 0.3534 |
| Gender (male), N (%) | 43 (61) | 143 (64) | 0.6826 | 38 (60) | 35 (55) | 0.5922 |
| BMI, median (IQR) | 22.2 (20.4- 23.9) | 22.1 (20.1- 24.4) | 0.7636 | 22.6 (20.3- 24.2) | 22.6 (20.6- 25.2) | 0.5371 |
| Smoking (Yes), N (%) | 27 (39) | 117 (52) | 0.0425* | 24 (38) | 24 (38) | 1.0000 |
| Alcohol use history (Yes), N (%) | 28 (40) | 88 (39) | 0.9360 | 26 (41) | 22 (34) | 0.4652 |
| Steroid use (Yes), N (%) | 0 (0) | 6 (3) | 0.1656 | 0 (0) | 1 (2) | 0.3154 |
| Diabetes mellitus (Yes), N (%) | 20 (29) | 53 (24) | 0.4175 | 17 (27) | 14 (22) | 0.5359 |
| ASA-PS (1/2/3), N (%) | 8 (11)/ 53 (76)/ 9 (13) | 3 (1)/ 207 (93)/ 13 (6) | <0.0001* | 8 (13)/ 48 (75)/ 8 (13) | 3 (5)/ 51 (80)/ 10 (15) | 0.2745 |
| Diagnosis (Pancreatic cancer), N (%) | 35 (50) | 111 (50) | 0.9739 | 32 (50) | 37 (58) | 0.3753 |
| Preoperative Cholangitis/Cholecystitis (Yes), N (%) | 13 (19) | 84 (37) | 0.0031* | 12 (19) | 15 (23) | 0.5157 |
| Duration of preoperative biliary drainage, median (IQR) | 38.5 (24- 62) | 48 (32- 89) | 0.0067* | 39.5 (24- 60.5) | 43 (27- 72.75) | 0.2536 |
| Laboratory data |  |  |  |  |  |  |
| HbA1c, median (IQR) | 5.7 (5.2- 6.4) | 5.8 (5.4- 6.3) | 0.4333 | 5.7 (5.1- 6.3) | 5.9 (5.5- 6.3) | 0.1371 |
| mGPS (0/1/2), N (%) | 37 (53)/ 26 (37)/ 7 (10) | 116 (52)/ 71 (32)/ 36 (16) | 0.4005 | 34 (53)/ 25 (39)/ 5 (8) | 36 (56)/ 19 (30)/ 9 (14) | 0.3646 |
| PNI, median (IQR) | 45.3 (40- 49) | 42.8 (39.1- 48.0) | 0.1431 | 45.6 (40.5- 49.7) | 43.4 (39.6- 48.8) | 0.3067 |
| Operative variables |  |  |  |  |  |  |
| Operation time, median (IQR) | 431.5 (347- 475) | 374 (325- 432) | 0.0002* | 418 (343.3- 467.3) | 413.5 (348.3- 462.8) | 0.9981 |
| Blood loss, median (IQR) | 400 (263- 963) | 220 (115- 405) | <0.0001* | 392.5 (240.3- 825) | 337.5 (171.3- 773.8) | 0.1744 |
| Transfusion (Yes), N (%) | 13 (19) | 8 (4) | <0.0001* | 8 (13) | 7 (11) | 0.7835 |
| Portal vein resection (Yes), N (%) | 20 (29) | 50 (22) | 0.2925 | 16 (25) | 21 (33) | 0.3296 |
| Main pancreatic duct, median (IQR), N (%) | 3 (2- 4) | 3 (2- 4) | 0.5078 | 3 (2- 4) | 3 (2- 4) | 0.4652 |
| Pancreatic texture (Soft), N (%) | 27 (39) | 111 (50) | 0.1013 | 26 (41) | 27 (42) | 0.8576 |
| Drain fluid AMY level in POD1 (IU/L), median (IQR) | 463 (98.5- 1821) | 1074 (162- 3338) | 0.0416* | 463 (101.5- 1936.5) | 795 (81- 5419.8) | 0.5246 |
| **Post operative complications** |  |  |  |  |  |  |
| All SSI, N (%) | 23 (33) | 30 (13) | 0.0002* | 22 (34) | 9 (14) | 0.0073* |
| Organ/Space SSI, N (%) | 19 (27) | 24 (11) | 0.0007* | 19 (30) | 6 (9) | 0.0038* |
| Incisional SSI, N (%) | 13 (19) | 8 (4) | <0.0001* | 12 (19) | 4 (6) | 0.0325* |
| Superficial incisional SSI, N (%) | 9 (13) | 8 (4) | 0.0038* | 9 (14) | 4 (6) | 0.1435 |
| Deep incisional SSI, N (%) | 4 (6) | 2 (1) | 0.0130* | 4 (6) | 1 (2) | 0.1711 |
| Severe Complications (CD grade Ⅲ or more), N (%) | 18 (26) | 23 (10) | 0.0012* | 18 (28) | 8 (13) | 0.0280* |
| Pancreatic fistula (Grade B or more), N (%) | 16 (23) | 14 (6) | <0.0001* | 16 (25) | 4 (6) | 0.0035* |
| Intra‐abdominal abscess, N (%) | 19 (27) | 22 (10) | 0.0003* | 21 (27) | 10 (13) | 0.0433* |
| Post‐pancreatectomy hemorrhage, N (%) | 4 (6) | 4 (2) | 0.0791 | 4 (5) | 2 (3) | 0.6812 |
| Delayed gastric emptying, N (%) | 4 (6) | 13 (6) | 0.9713 | 4 (6) | 6 (9) | 0.5101 |
| Percutaneous drainage, N (%) | 16 (23) | 20 (9) | 0.0020* | 16 (25) | 6 (9) | 0.0191* |
| Reoperation, N (%) | 2 (3) | 3 (1) | 0.3942 | 2 (3) | 2 (3) | 1.0000 |
| Mortality, N (%) | 1 (1) | 0 (0) | 0.0738 | 1 (2) | 0 (0) | 0.3154 |

*BMI,* body mass index; *ASA-PS,* American Society of Anesthesiology physical status; *mGPS,* **modified Glasgow Prognostic Score;** *PNI,* **Prognostic Nutritional Index; *SSI,* Surgical Site infection.**

* Statistically significant
