# Supplementary material for: Prolonged Prophylactic Antibiotics Based on Preoperative Bile Culture Reduce Surgical Site Infections After Pancreaticoduodenectomy Following Preoperative Biliary Drainage: A Propensity‐Matched Analysis
Source: Ann Gastroenterol Surg. 2025 Aug 14;10(1):197–210. doi: 10.1002/ags3.70076 (PMC12757157; doi:10.1002/ags3.70076)
Supplement: Supplementary file 6 — Table S3: Risk factors of postoperative pancreatic fistula after pancreaticoduodenectomy. [file AGS3-10-197-s001.docx]

**Supplementary Table 3**

Risk factors of postoperative pancreatic fistula after pancreaticoduodenectomy

|  |  |  | Univariate analysis |  |  |  | Multivariate analysis |  |  |
| --- | --- | --- | --- | --- | --- | --- | --- | --- | --- |
|  | POPF (n=38) (11%) | No POPF (n=314) (89%) | OR | 95% CI | *p* value |  | OR | 95% CI | *p* value |
| Age (≥75), n (%) | 13 (34) | 98 (31) | 1.15 | 0.56-2.33 | 0.7070 |  |  |  |  |
| Gender (male), n (%) | 30 (79) | 189 (60) | 2.48 | 1.10-5.59 | 0.0243* |  | 1.35 | 0.47-3.85 | 0.57816 |
| BMI (≥25), n (%) | 12 (32) | 52 (17) | 2.33 | 1.10-4.90 | 0.0234* |  | 2.81 | 1.12-7.87 | 0.03130* |
| Smoking (Yes), n (%) | 19 (50) | 154 (49) | 1.04 | 0.53-2.04 | 0.9114 |  |  |  |  |
| Alcohol use history (Yes), n (%) | 21 (55) | 118 (38) | 2.05 | 1.04-4.05 | 0.0352* |  | 1.80 | 0.72-4.50 | 0.20438 |
| Steroid use (Yes), n (%) | 1 (3) | 5 (2) | 1.67 | 0.19-14.69 | 0.6402 |  |  |  |  |
| Diabetes mellitus (Yes), n (%) | 5 (13) | 80 (25) | 0.44 | 0.17-1.17 | 0.0937 |  |  |  |  |
| ASA-PS (≥3), n (%) |  |  |  |  |  |  |  |  |  |
| Diagnosis (Pancreatic cancer), n (%) | 8 (21) | 161 (51) | 0.25 | 0.11-0.57 | 0.0004* |  | 1.19 | 0.33-4.20 | 0.79175 |
| Preoperative Cholangitis/Cholecystitis (Yes), n (%) | 11 (29) | 99 (32) | 0.88 | 0.42-1.86 | 0.7458 |  |  |  |  |
| Type of initial biliary drainage (Internal), n (%) | 30 (79) | 263 (84) | 1.38 | 0.60-3.17 | 0.4534 |  |  |  |  |
| Duration of preoperative biliary drainage (≥45 days), n (%) | 17 (45) | 156 (50) | 0.82 | 0.42-1.61 | 0.5647 |  |  |  |  |
| Laboratory data |  |  |  |  |  |  |  |  |  |
| HbA1c (≥6.5%), n (%) | 5 (13) | 75 (24) | 0.48 | 0.18-1.28 | 0.1361 |  |  |  |  |
| Albumin (≥3.5 g/dL), n (%) | 23 (61) | 170 (54) | 0.77 | 0.39-1.53 | 0.4550 |  |  |  |  |
| mGPS (≥2), n (%) | 5 (13) | 47 (15) | 1.16 | 0.43-3.13 | 0.7664 |  |  |  |  |
| Operation time (≥399 min: average), n (%) | 20 (53) | 144 (46) | 1.31 | 0.67-2.57 | 0.4293 |  |  |  |  |
| Blood loss (≥444 mL: average), n (%) | 15 (39) | 100 (32) | 1.40 | 0.70-2.79 | 0.3428 |  |  |  |  |
| Transfusion (Yes), n (%) | 4 (11) | 28 (9) | 0.83 | 0.28-2.54 | 0.7445 |  |  |  |  |
| Pancreatic texture (Soft), n (%) | 42 (66) | 125 (43) | 5.80 | 2.48-13.56 | <0.0001* |  | 2.78 | 0.74-10.45 | 0.12061 |
| Main pancreatic duct ≤3 mm, n (%) | 25 (66) | 113 (36) | 3.42 | 1.68-6.95 | 0.0004* |  | 0.98 | 0.40-2.43 | 0.97212 |
| Drain fluid AMY level in POD1 >4000 IU/L, n (%) | 26 (68) | 52 (17) | 10.92 | 5.18-23.02 | <0.0001* |  | 11.28 | 4.30-29.61 | <0.0001* |
| Prophylactic antibiotic for 3 days, n (%) | 16 (42) | 224 (71) | 0.29 | 0.15-0.58 | 0.0003* |  | 0.16 | 0.07-0.37 | 0.00001* |

* Statistically significant
